# Beyond the check-up: how well-child exams, pediatric specialists, and provider recommendations can close HPV vaccine gaps for Chinese American Teens

**DOI:** 10.1007/s10552-025-02110-3

**Published:** 2026-01-17

**Authors:** Lin Zhu, Xinrui Li, Elaine Zhiqing Liu, Philip T. Siu, Shumenghui Zhai, Chun Pan, Nikki Cao, Grace X. Ma

**Affiliations:** 1 Center for Asian Health, Lewis Katz School of Medicine, Temple University, Philadelphia, PA USA; 2 Department of Urban Health and Population Science, Lewis Katz School of Medicine, Temple University, Philadelphia, PA USA; 3 Psychology and Human Development Department, Peabody College, Vanderbilt University, Nashville, TN USA; 4 Esperanza Health Center, Philadelphia, PA USA; 5 Department of Mathematics and Statistics, Hunter College, New York, NY USA; 6 School of Nursing, Pacific Lutheran University, Tacoma, WA USA

**Keywords:** Human papillomavirus vaccine, Chinese American, Multilevel factors, Disaggregated data, Adolescents

## Abstract

**Purpose:**

This study aimed to identify provider- and practice-level factors influencing human papillomavirus (HPV) vaccine uptake among Chinese American (CA) adolescents. Despite increasing public health efforts, HPV vaccination rates in this population remain suboptimal, and knowledge on the link between provider/practice characteristics and vaccine uptake is limited.

**Methods:**

We analyzed data from 1,272 CA adolescents (aged 13–17) using the National Immunization Survey-Teen (2015–2019) datasets. Key measures included HPV vaccine initiation and completion (based on proxy reporting and verification), accompanied by various multilevel factors: provider-level (recommendation, specialty), practice-level (facility type, vaccine acquisition, well-child exam), parent-level (mother’s education, marital status), and adolescent-level characteristics. Weighted descriptive statistics and stepwise multivariate logistic regression were used to examine associations.

**Results:**

Overall, 71.22% of CA adolescents initiated at least one HPV vaccine dose, while 48.14% completed the regimen. Provider's recommendation (initiation OR = 21.50, completion OR = 8.12), having a pediatrician (initiation OR = 5.37, completion OR = 3.20), and receiving the 11–12-year-old well-child exams significantly predict both initiation and completion. Regional disparities were observed, with adolescents in the Northeast and West showing higher completion rates than those in the South. Unexpectedly, adolescents not enrolled in school and those with mothers who had less than 12 years of education or were unmarried showed higher completion rates.

**Conclusion:**

Provider recommendations and pediatric care are crucial for improving HPV vaccination rates among CA adolescents. Addressing regional disparities and implementing targeted interventions, including enhanced provider training focused on culturally sensitive communication, are essential to dismantle systemic barriers and improve comprehensive vaccination coverage in this underserved population.

## Introduction

Sexually transmitted infections (STIs) pose a significant public health challenge, with rates steadily increasing since 2019 [1]. Human papillomavirus (HPV) is the most common form of sexually transmitted disease in the United States, affecting 42 million people [2]. HPV is a leading cause of six cancers—anal, cervical, oropharyngeal, penile, vaginal, and vulvar—causing approximately 36,000 cases annually in the United States [3]. Among these, cervical cancer has historically been the most common and a leading cause of cancer deaths in women. Importantly, HPV-related cancers are largely preventable. Current vaccines show over 90% efficacy against HPV-associated cancers and genital warts [3]. To optimize the preventive effects, the Advisory Committee on Immunization Practices (ACIP) recommends routine vaccination at ages 11–12, before most sexual exposure occurs [4].

Asian Americans are one of the fastest-growing demographic groups, currently comprising 7% of the population (twenty-four million) and projected to quadruple by 2060 [5]. Although Asian Americans comprise over 20 distinct ethnicities with diverse cultural, linguistic, and social backgrounds, research often treats them as a homogeneous group, masking subgroup disparities [6–8]. These disparities remain understudied [9]. Chinese Americans (CA) are the largest Asian subgroup (21.66%), yet recent research on their health disparities is limited [10]. CAs are disproportionately affected by cervical cancer and several other HPV-related cancers [11–13]. However, our understanding of their HPV vaccine uptake behaviors is limited.

According to the National Health Interview Survey from 2006 to 2018, less than half of the CA population completed their HPV, well below the Healthy People 2030 goal of 80% HPV vaccine uptake initiated by the U.S. Department of Health and Human Services [14, 15]. Previous studies have found low vaccine awareness and low vaccine initiation and completion among CA adolescents [16, 17]. Developing targeted interventions to address these disparities requires an understanding of the multilevel factors affecting HPV vaccine uptake. Prior studies have primarily examined parent-level characteristics (e.g., income, education, and health insurance status), which significantly influence HPV vaccine uptake among CA adolescents [17, 18]. Additional determinants such as HPV vaccine knowledge, perceived safety, and disease susceptibility have also been identified as crucial factors [19–21]. Beyond individual and parental influences, research has also suggested associations between vaccination rates and provider recommendations, practice-level factors, and structural barriers (e.g., lack of access) in the general population [22–24]. The importance of provider- and practice-level characteristics in vaccine uptake remain understudied in the CA community.

This study aims to address this gap by examining disaggregated data on CA adolescents. To our knowledge, this will be the first to utilize a disaggregated nationally representative sample of CA adolescents to investigate the combined influence of multilevel factors, particularly provider- and practice-level characteristics, on HPV vaccine uptake. By analyzing these factors, this study will provide comprehensive insights to inform targeted public health interventions for improving vaccination rates in this population.

## Methods

### Data

This study included data from the National Immunization Survey-Teen (NIS-Teen) collected between 2015 and 2019. The NIS-teen is an ongoing survey supported by the CDC and conducted by the National Opinion Research Center (NORC). NIS-Teen collects demographic information and HPV vaccination status from parents or guardians of adolescents aged 13–17 through household surveys. The household surveys collect data through telephone interviews with parents or guardians in all 50 states, the District of Columbia, and some U.S. territories. Cell phone numbers are randomly selected and called to enroll one or more age-eligible teens from the household. During the call, the parents and guardians of eligible teens are asked for the names of their children’s vaccination providers and permission to contact them.

Having received verbal consent from parents or guardians, a questionnaire is sent via mail to the adolescents’ immunization providers to request information on the types of vaccinations, number of doses, dates of administration, and other administrative data about the health care facility [25]. For simplicity, we’ll refer to the parent or guardian interviewed as the adolescent’s parent. Population-level weights are used to account for the multi-level sampling design used and the methods and weighting procedures for NIS-Teen were described previously [26].

## Measures

*HPV vaccine initiation and completion.* HPV vaccine uptake status was determined using data from the provider survey. We defined HPV vaccination initiation as a binary variable (yes/no), with “yes” indicating that the adolescent had received at least one dose of the HPV vaccine, according to the provider-reported number of HPV vaccine doses administered by the time of the interview. HPV vaccination completion was also defined as a binary variable (yes/no), based on the age at initiation and the number of doses received. Specifically, completion was defined as receiving two doses if the vaccine series was initiated before the age of 15 or three doses if the series was initiated at or after the age of 15, in accordance with the recommended HPV vaccination schedule [2].

*Provider- and practice-level factors*. This study examined two provider-level factors and three practice-level factors. The provider-level factors were: (1) whether the adolescents received a recommendation for the HPV vaccine from their providers (binary variable: yes/no), reported by adolescents/parents, and (2) the provider's specialty, which was categorized into six groups: pediatrics, family practice, general practice, internal medicine, obstetrics/gynecology (OB/GYN), and other. The three practice-level factors were as follows: Firstly, the types of healthcare facilities were categorized into all public, all private, all hospital facilities, all specialized clinics (e.g., STD/school/teen clinics), mixed, and others. Secondly, vaccine acquisition source was determined by whether the practices ordered HPV vaccines from the state/local health department through the Vaccine for Children (VFC) program (i.e., yes/no). Lastly, practices reported whether they conducted the 11–12-year-old well child exam or check-up for adolescents (i.e., yes/no).

*Parent-level factors.* We examined two sociodemographic characteristics of the parents, obtained from the household survey. The first characteristic was the mother's educational level, which was categorized into four groups: less than 12 years of education, 12 years of education, more than 12 years of education but not a college graduate, and college graduate. The second characteristic was the mother's marital status, which was categorized into two groups: currently married and not currently married.

*Adolescent-level factors*. We also examined seven characteristics of adolescents from the household survey. Sex was categorized as male or female. The Census region of residence was categorized into four groups: Northeast, Midwest, South, and West. Current grade of the adolescents was categorized into three groups: 6th to 8th grade, 9th to 12th grade, and high school (HS)/general educational development (GED)/not in school. Health insurance status was categorized into four groups: private insurance only, any Medicaid, other insurance, and uninsured. “Other insurance” includes the Children’s Health Insurance Program, military insurance, Indian Health Service, and any other type of health insurance otherwise not mentioned. Household poverty level was categorized into three groups: above poverty level and > $75 k, above poverty level <  = $75 k, and below poverty level. Nativity status was categorized into two groups: whether the adolescent was born in the US or not.

## Statistical analysis

This study included 1,272 CA adolescents who had sufficient provider data to determine vaccination status. To ensure that the sampled individuals were representative of the U.S. population demographics, we followed the instructions provided in the NIS-Teen User’s Guide and applied the appropriate weights and stratum variables [25]. This adjustment accounted for non-response, unresolved telephone numbers, missing provider data, and the complex survey design. When calculating average estimates over multiple years, we combined the NIS-Teen data from the relevant years and divided the annual weights by the number of years included in the analysis, as required by the NIS-Teen User's Guide for multi-year data amalgamation [25]. Weighted analyses were conducted for all statistical tests. Weighted descriptive statistics, including percentages and 95% confidence intervals (CIs), were presented for various adolescent-, parent-, provider-, and practice-level characteristics of the CA sample. For comparison purposes, descriptive statistics for the aggregate Asian sample, CA subsample included, were also provided. Stepwise multivariate logistic regression was employed to examine the multilevel factors associated with HPV vaccine initiation and completion, and the odds ratios (ORs) with 95% CIs were reported. Variables were first examined individually in univariate logistic regression analyses, and those with *p* < 0.05 were retained for inclusion in multivariable logistic regression models examining HPV vaccine initiation and completion. All statistical analyses were performed using Stata version 16, with the *svy* command used to apply weighting and the *subpop* option used for subpopulation analyses [27]. Statistical significance was determined at a *p*-value < 0.05. Figures are generated in RStudio (version 4.4.2) with packages dplyr (version 1.1.4), tidyr (version 1.3.1), ggplot2 (version 3.5.1), ggtext (version 0.1.2), and scales (version 1.3.0) [28].

## Results

The weighted socioeconomic and immigration-related characteristics of the adolescents and parents of the CA adolescents and the aggregate Asian American adolescent sample are presented in Table 1. Among the study sample, the mean age was 14.96 years and consisted of 37.49% boys and 62.51% girls. 21.72% of the adolescents had a family income of $75 K or less, with 3.24% without any health insurance. Moreover, 12.76% of CA adolescents were born outside of the US. With regards to parental socioeconomic status, 18.24% of the CA adolescents reported that their mothers were not married at the time of the survey, and about three quarters (75.43%) had mothers with a college degree. The CA sample varied significantly from the aggregate AA sample in several characteristics. The proportion of CA adolescents reside in West (41.82%) was higher than the AA adolescents (36.19%). A higher percentage of CA adolescents had private insurance coverage (75.47%) compared to AA adolescents (65.09%). CA adolescents also had a higher proportion of household incomes above $75,000 and mothers who were college graduates compared to AA adolescents. Conversely, AA adolescents had a higher proportion of Medicaid coverage than CA adolescents.

**Table 1:** Weighted descriptive statistics of characteristics: sociodemographic and immigration in adolescents and parents, and service delivery in providers/practices (NIS-Teen 2015–2019)

Proportion or mean (95% CI)	CA (*n* = 1,272)	Aggregate AA (*n* = 5,321)
Age (mean)	14.9586 (14.7814, 15.1357)	14.99 (14.9116, 15.0684)
Sex		
Male	0.3749 (0.3483, 0.4015)	0.4971 (0.4837, 0.5105)
Female	0.6251 (0.5985, 0.6517)	0.5029 (0.4837, 0.5105)
Census region of residence		
Northeast	0.2337 (0.2104, 0.2570)	0.2052 (0.1943, 0.2161)
Midwest	0.1413 (0.1222, 0.1604)	0.1552 (0.1455, 0.1649)
South	0.2068 (0.1845, 0.2291)	0.2778 (0.2658, 0.2898)
West	**0.4182 (0.3911, 0.4453)**	**0.3619 (0.3490, 0.3748)**
Current grade		
6th—8th	0.2754 (0.2509, 0.2999)	0.2655 (0.2536, 0.2774)
9th to 12th	0.7181 (0.6934, 0.7428)	0.7225 (0.7105, 0.7345)
HG/GED/not in school	0.0065 (0.0021, 0.0109)	0.0121 (0.0092, 0.0150)
Insurance		
Private insurance only	**0.7547 (0.7311, 0.7783)**	**0.6509 (0.6381, 0.6637)**
Any Medicaid	**0.1503 (0.1307, 0.1699)**	**0.2383 (0.2269, 0.2497)**
Other insurance	0.0626 (0.0493, 0.0759)	0.0726 (0.0656, 0.0796)
Uninsured	0.0324 (0.0227, 0.0421)	0.0382 (0.0330, 0.0434)
Household income poverty level		
Above poverty > $75 k	**0.7063 (0.6813, 0.7313)**	**0.5929 (0.5797, 0.6061)**
Above poverty < = $75 k	0.2172 (0.1945, 0.2399)	0.2575 (0.2458, 0.2692)
Below poverty	0.0764 (0.0618, 0.0910)	0.1496 (0.1400, 0.1592)
Nativity status		
US-born	0.8724 (0.8541, 0.8907)	0.8119 (0.8014, 0.8224)
Not US-born	0.1276 (0.1093, 0.1459)	0.1881 (0.1776, 0.1986)
Marital status of mother		
Married	0.8176 (0.7964, 0.8388)	0.805 (0.7944, 0.8156)
Not currently married	0.1824 (0.1612, 0.2036)	0.195 (0.1844, 0.2056)
Education of mother		
< 12 years	0.0505 (0.0385, 0.0625)	0.0897 (0.0820, 0.0974)
12 years	0.0902 (0.0745, 0.1059)	0.1565 (0.1467, 0.1662)
> 12 years, non-college grad	0.1049 (0.0881, 0.1217)	0.1325 (0.1234, 0.1416)
College graduate	**0.7543 (0.7306, 0.7780)**	**0.6214 (0.6084, 0.6344)**
Provider recommendation for HPV vaccine		
Yes	**0.8190 (0.7978, 0.8402)**	**0.7309 (0.7190, 0.7428)**
No	**0.1810 (0.1598, 0.2022)**	**0.2691 (0.2572, 0.2810)**
Type of facility for teens providers		
All public facilities	0.0885 (0.0729, 0.1041)	0.1003 (0.0922, 0.1084)
All private facilities	0.0814 (0.0664, 0.0964)	0.0874 (0.0798, 0.0950)
All hospital facilities	0.6262 (0.5996, 0.6528)	0.6394 (0.6265, 0.6523)
All STD/school/teen clinics or other facilities	0.0487 (0.0369, 0.0605)	0.0391 (0.0339, 0.0443)
Mixed	0.1552 (0.1353, 0.1751)	0.1338 (0.1247, 0.1429)
Specialty of provider		
Pediatrics	0.7520 (0.7283, 0.7757)	0.7454 (0.7337, 0.7571)
Family practice	0.2880 (0.2631, 0.3129)	0.2928 (0.2806, 0.3050)
General practice	0.1614 (0.1412, 0.1816)	0.1371 (0.1279, 0.1463)
Internal medicine	0.2012 (0.1792, 0.2232)	0.1736 (0.1634, 0.1838)
OB/GYN	0.1503 (0.1307, 0.1699)	0.1291 (0.1201, 0.1381)
Other	0.1303 (0.1118, 0.1488)	0.1540 (0.1443, 0.1637)
Whether provider ordered vaccines from state/local health dept		
Some or all	0.8147 (0.7933, 0.8361)	0.8188 (0.8085, 0.8292)
No	0.1853 (0.1639, 0.2067)	0.1812 (0.1709, 0.1916)
Whether provider conducted 11–12-year-old well child exam to teen		
Yes	**0.9594 (0.9486, 0.9702)**	**0.9049 (0.8970, 0.9128)**
No	**0.0406 (0.0298, 0.0514)**	**0.0951 (0.0872, 0.1030)**
Bold: *p* < 0.05		

Table 1 also presents the weighted provider- and practice-level characteristics for the CA sample and the aggregated Asian American sample. Among CA adolescents, 81.9% received a recommendation for the HPV vaccine from their providers. Over half 62.62% reported hospitals as the only types of facilities where their providers were located. The majority (75.20%) of the CA adolescents had a pediatrician, 28.80% had a family practitioner, while 20.12% had an internalist. About four out of five (81.47%) in the CA sample had a provider who ordered vaccines from state or local health departments. In addition, 95.94% of the CA adolescents reported receiving the 11–12-year-old well child exam from their providers. We noted significant variations between the CA and aggregate AA sample on several factors, including providers' recommendations for the HPV vaccine and whether the adolescents received an 11–12-year-old well child exam. Specifically, CA adolescents were more likely to receive a recommendation for the HPV vaccine and to have had an 11–12-year-old well child exam compared to the aggregated AA adolescents.

**Fig. 1:**
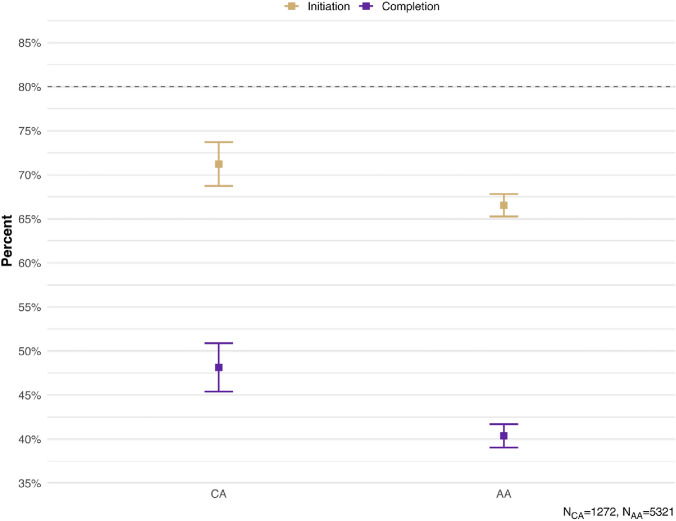
HPV vaccination intiation and completion rate in CA and aggregate AA adolescents, NIS-Teen 2015–2019

Figure 1 present the weighted HPV vaccination initiation and completion rates, as recorded in the providers’ records. Based on the provider reports, only about two thirds (71.22%) of the CA adolescents received at least one shot, while about half (51.86%) did not complete their HPV vaccine regimen. Vaccine uptake was even lower according to household- reported data with only half (51.52%) initiating the vaccine and one in five (21.85%) completing the HPV vaccine regimen (results not included). In addition, “not receiving a recommendation from the provider” was cited by 25.17% of the unvaccinated CA adolescents as a reason for not receiving any HPV vaccines. This was closed 24.19% rate in the aggregated AA adolescents. Other reasons were examined, but the results were not presented because of low case counts, in compliance with the NIS-Teen data release policy.

**Fig. 2:**
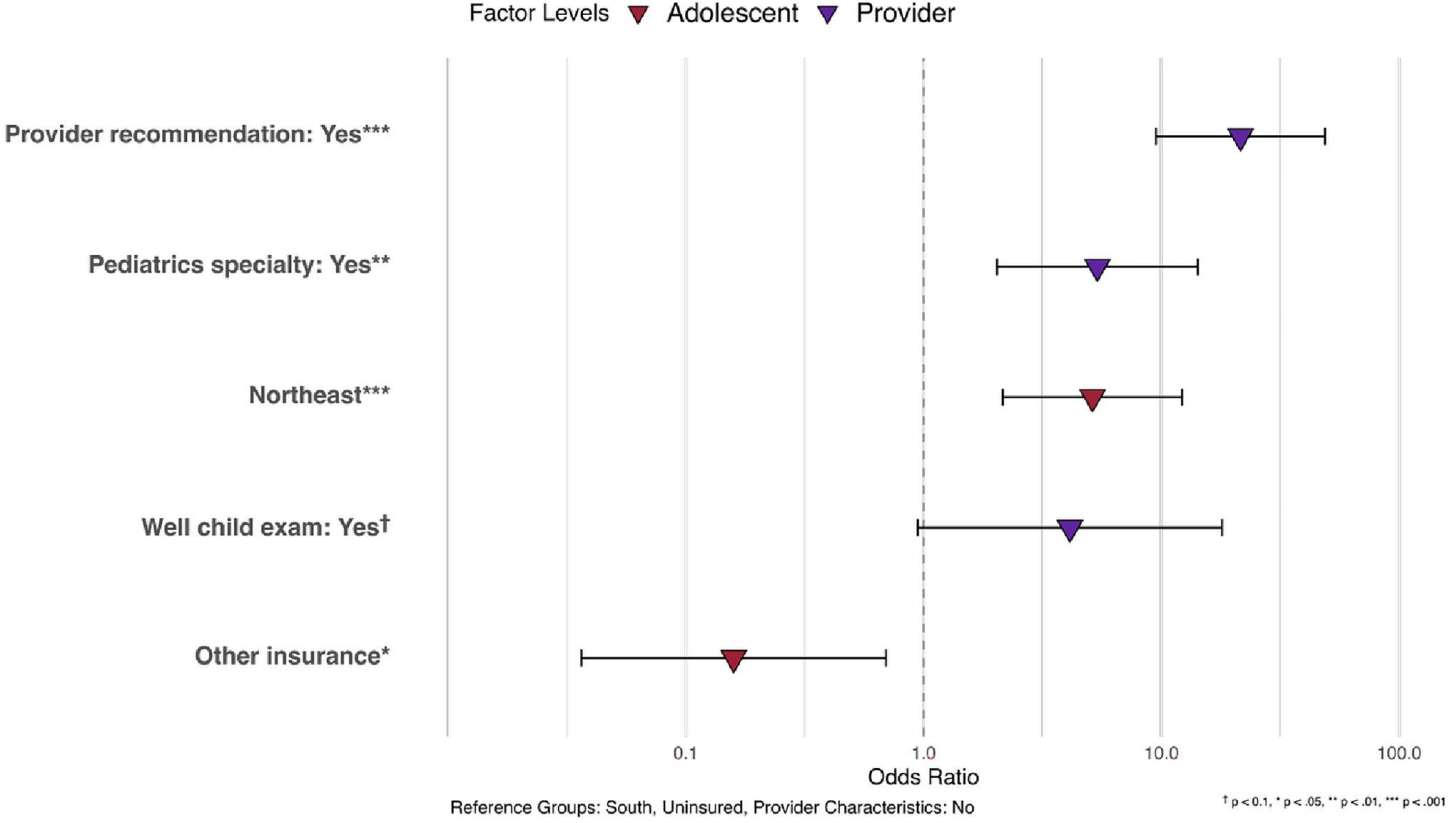
Association between multilevel factors and provider reported HPV vaccine initiation in CA adolescents NIS-Teen 2015–2019 (*n* = 1,272)

Table 2 presents the results of the multivariate logistic regression on HPV vaccination initiation. We found that receiving a physician's recommendation for the HPV vaccine (OR = 21.50, 95%CI = 9.48–48.74) was significantly associated with a higher likelihood of initiating the HPV vaccine regimen. Having a pediatrician as a healthcare provider (OR = 5.37, 95% CI = 2.04–14.18) was a significant predictor of HPV vaccination initiation. Additionally, adolescents who received an 11–12-year-old well child exam were more likely to initiate HPV vaccination, although this association was only marginally significant (OR = 4.12, 95% CI = 0.94–17.97). In addition, our results showed that CA adolescents living in the Northeast was more likely than those living in the South to initiate HPV vaccination (OR = 5.13, 95%CI = 2.15–12.22) Fig. 2.

**Table 2:** Logistic regression results of provider reported HPV initiation in CA adolescents, odds ratio and 95% confidence interval, NIS-Teen 2015–2019

	Odds Ratio (95%CI), *p*-value
*Adolescent-level characteristics*	
Sex	
Male	Reference
Female	0.9141 (0.4893, 1.7079), 0.7780
Census region of residence	
Northeast	5.1285 (2.1526, 12.2185), < 0.001***
Midwest	1.7274 (0.7387, 4.0395), 0.207
South	Reference
West	1.6102 (0.7115, 3.6439), 0.253
Insurance	
Private insurance only	1.0421 (0.3033, 3.5803), 0.948
Any Medicaid	2.9675 (0.7224, 12.1903), 0.131
Other insurance	0.1591 (0.0364 0.6951), 0.015**
Uninsured	Reference
*Provider-/practice-level characteristics*	
Provider recommendation of HPV vaccine (ref: no)	21.5026 (9.4850, 48.7472), < 0.001***
Pediatrics specialty (ref: no)	5.374121 (2.0360, 14.1849), 0.001**
Provider ordered vaccines from state/local health dept (ref: no)	0.92730 (0.3957, 2.1730), 0.862
Provider conducted 11–12-year-old well child exam to teen	4.1207 (0.9448, 17.9722), 0.060^†^

^†^ *p* < 0.1, * *p* < 0.05, ** *p* < 0.01, *** *p* < 0.001

**Fig. 3:**
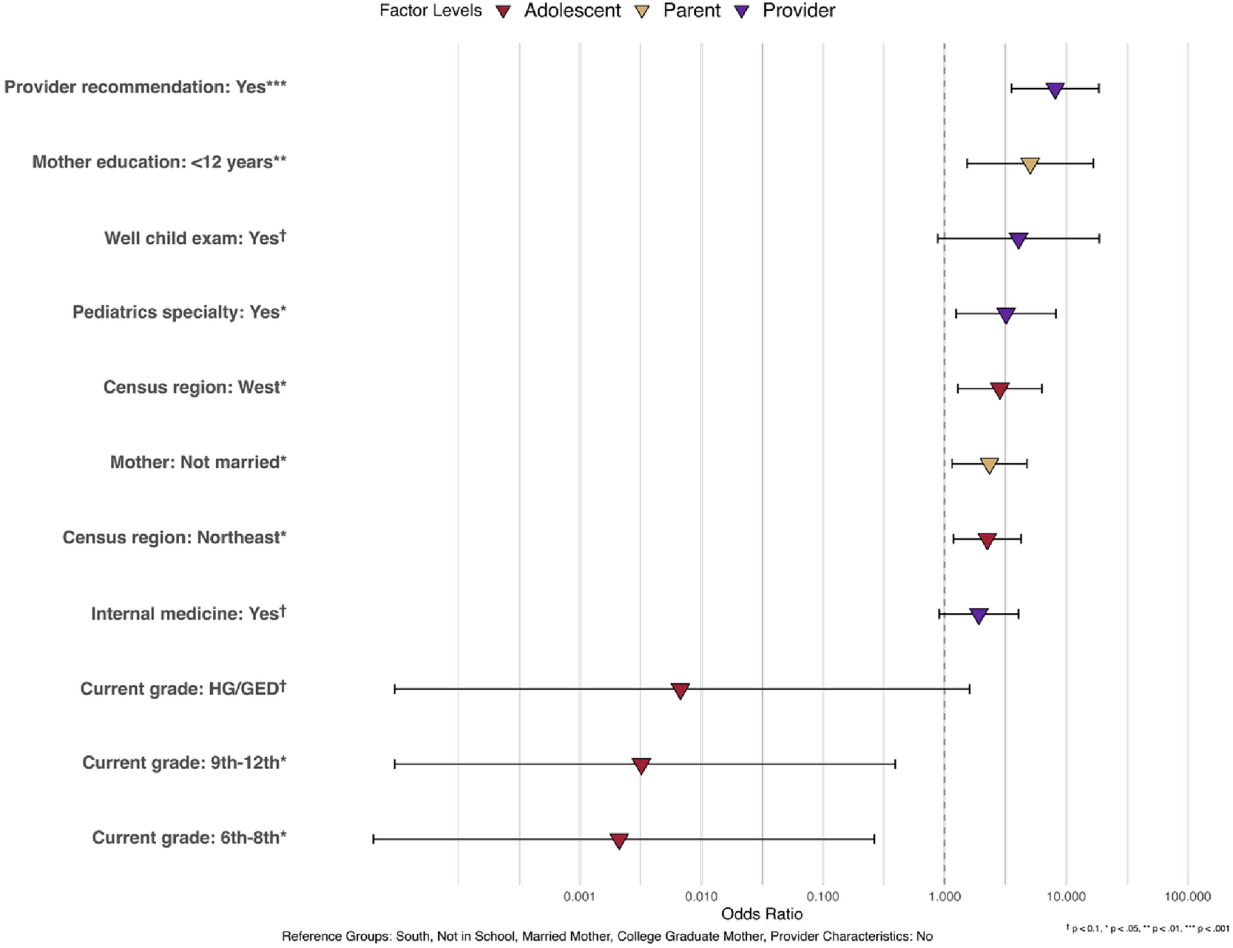
Association between multilevel factors and provider reported HPV vaccine completion in CA adolescents, NIS-Teens 2015–2019 (*n* = 1,272)

Table 3 presents the association of various factors with HPV vaccination completion in CA adolescents. Having a provider recommendation (OR = 8.12, 95% CI = 3.54 – 18.60) and having a pediatrician (OR = 3.20, 95% CI = 1.24 – 8.22) were both significant predictors of completing the HPV vaccine regimen. Additionally, living in the West (OR = 2.84, 95% CI = 1.28–6.32) or Northeast (OR = 2.24, 95% CI = 1.18–4.25) was significantly associated with higher HPV vaccination completion compared with adolescents living in the South. Adolescents with mothers who are not currently married have a higher likelihood of HPV vaccination completion (OR = 2.34, 95% CI = 1.15 – 4.75). Surprisingly, CA adolescents in 6th-8th grade (OR = 0.0021, 95% CI = 0.00–0.26) and those in 9th-12th grade (OR = 0.0032, 95% CI = 0.00–0.39) were significantly less likely to complete the HPV vaccine regimen compared to those who do not currently enroll in school. Moreover, having mothers with less than 12 years of education was significantly associated with higher HPV completion likelihood compared to having mothers of college graduates (OR = 5.06, 95% CI = 1.53 – 16.73) (Fig. 3)

**Table 3:** Logistic regression results of provider reported HPV completion in CA adolescents, odds ratio and 95% confidence interval (NIS-Teen 2015–2019**)**

*Adolescent-level characteristics*	Odds Ratio (95%CI), *p*-value
Sex	
Male	Reference
Female	1.29223 (0.7274, 2.2957), 0.382
Census region of residence	
Northeast	2.2422 (1.1829, 4.2503), 0.013**
Midwest	1.2750 (0.6362, 2.5551), 0.493
South	Reference
West	2.8449 (1.2816, 6.3153), 0.010**
Current grade	
6th– 8th	0.0021 (0.00002, 0.2643), 0.012**
9th to 12th	0.0032 (0.00003, 0.3920), 0.019**
HG/GED	0.0067 (0.00003, 1.6019), 0.073^†^
Not in school	Reference
Insurance	
Private insurance only	2.7240 (0.5440, 13.64145), 0.223
Any Medicaid	3.1345 (6372, 15.4188), 0.160
Other insurance	0.5326 (0.0850, 3.3362), 0.501
Uninsured	Reference
*Parent-level characteristics*	
Marital status of mother	
Currently married	Reference
Not currently married	2.3360 (1.1489, 4.7495), 0.019**
Education of mother	
< 12 years	5.0600 (1.5304, 16.7295), 0.008**
12 years	1.9556 (0.6636, 5.7634), 0.224
> 12 years, non-college grad	0.4836 (0.1716, 1.3626), 0.169
College graduate	Reference
*Provider/practice-level characteristics*	
Provider recommendation of HPV vaccine (ref: no)	8.1190 (3.5433, 18.6036), < 0.001***
Pediatrics specialty (ref: no)	3.1996 (1.2447, 8.2245), 0.016**
Internal medicine (ref: no)	1.9101 (0.9015, 4.0468), 0.091^†^
Provider ordered vaccines from state/local health dept (ref: no)	1.3713 (0.6424, 2.9272), 0.414
Provider conducted 11–12-year-old well child exam to teen	4.0555 (0.8806, 18.6781), 0.072^†^

^†^ *p* < 0.1, * *p* < 0.05, ** *p* < 0.01, *** *p* < 0.001

## Discussion

To the best of our knowledge, this study is one of the first to apply a nationally representative sample to examine HPV vaccine uptake and to investigate practice- and provider-level factors influencing HPV vaccine uptake among CA adolescents. The CA is the largest Asian American subgroup, accounting for approximately 22% of the Asian Americans [29, 30]. As a result of an aggregated multiple-year cross-sectional design, our findings provide a comprehensive snapshot of HPV vaccination patterns among CAs and support data-driven public health policies. Although CA adolescents show slightly higher HPV vaccine uptake than in aggregated Asian peers (71% vs. 67% initiation; 48% vs. 40% completion), these rates remain below the 80% benchmark set by Healthy People 2030 [15]. Additionally, our multilevel model revealed significant associations between provider, parent, and adolescent-level factors and HPV vaccine uptake.

Provider recommendations emerged as the strongest predictor of both initiating and completing the HPV vaccine series. This aligns with previous studies and reinforces the need for provider recommendations on HPV vaccination [31–33]. Notably, while 95.94% of CA adolescents attended well‑child exams, nearly 20% did not receive a recommendation, and 25% cited this as a primary barrier. This disconnect suggests that providers may not consistently prioritize HPV vaccine discussions during routine visits, even for this high-risk population [31, 34–36]. Our findings also highlight the need for provider training that emphasizes culturally attuned vaccine communications. While well-child exams showed marginal statistical significance, the consistent positive associations and substantial effect sizes suggest clinical importance that warrants further investigation with larger sample sizes.

Specialty also influenced vaccine uptake. Recommendations from pediatricians (and, to a lesser extent, internists) were significantly associated with higher uptake. In contrast, providers in family medicine, OB/GYN, and general practice may be less likely to promote HPV vaccination due to training gaps or role differences [34, 36–39].

To address this disparity, mandatory HPV vaccine competency training for all primary care specialties through continuing medical education (CME) programs [40], electronic health record (EHR) prompts specifically for family medicine providers during adolescent visits [41], and cross-specialty reimbursement incentives are potential strategies to encourage broader HPV vaccine administration [42]. The exceptionally large effect size for provider recommendation does warrant cautious interpretation and may reflect several methodological limitations. This finding could indicate potential endogeneity, whereby adolescents were more likely to recall having received a recommendation from their providers when vaccination had already occurred, creating a circular relationship between the provider recommendation and vaccine uptake. Additionally, unmeasured confounding factors that influence both provider recommendation patterns and vaccination uptake could inflate this association. Future research should examine longitudinal data to explore the temporal sequence of recommendation and vaccination to make causal inference.

Regional differences in healthcare access and resource allocation also emerged. Adolescents in the South (and the Midwest) had significantly lower HPV vaccine uptake compared to the Northeast and West. These differences may reflect varying policies, provider availabilities, and socioeconomic factors [43–46]. Although the Vaccines for Children (VFC) program did not reach significance in our analysis, it remains a potentially valuable tool for low-income CA families. Future studies should evaluate how state-level infrastructure and outreach contribute to regional gaps.

Unexpectedly, we observed that CA adolescents not enrolled in school had higher odds of completing HPV vaccination compared to those attending traditional schools. The small number of adolescents not currently enrolled in school (< 0.65% of the sample, as shown in Table 1) likely contributed to the statistical instability of these findings, as evidenced by the extremely low odds ratios and wide confidence intervals for the school enrollment categories. Future research with larger sample sizes should better explore these associations and potential cohort or period effects. Furthermore, CA mothers with less than 12 years of education or those who were currently unmarried had significantly higher odds of their children completing the HPV vaccination series. These findings, which differed from general population trends, may reflect increased engagement with public health services such as the VFC program or differing levels of trust in healthcare messaging [47–49], which could increase awareness of free vaccination services and facilitate access through safety-net providers who may more actively promote HPV vaccination. Additionally, residual confounding from unmeasured variables such as healthcare utilization patterns, provider types, or community-level factors may partially explain these findings, highlighting the need for future studies to examine these pathways more directly. Future research is needed to explore these dynamics among CA subgroups.

Our study has several limitations. Firstly, the cross-sectional nature of the data restricted the identification of health determinants and the causal pathways. Longitudinal studies are needed to understand multilevel determinants of vaccine uptake. Using an existing set of survey measures also restricted the exploration of nuanced psychosocial factors and their impacts on vaccine uptake. Secondly, our data only included HPV uptake information from provider-reported data, which may underrepresent vaccine access from community pharmacies, which play a critical role in Asian American communities [50–52]. Building on the widespread accessibility of community pharmacies, particularly in underpriviledged communities and medically underserved regions, researchers have recognized their potential as strategic venues for improving HPV vaccination rates [53, 54]. Future studies should investigate pharmacy-based and culturally responsive interventions. Furthermore, while we did not formally test for multicollinearity, our stepwise regression approach helped mitigate potential issues by systematically including variables based on statistical significance. We acknowledge that the high correlation potentially present between variables such as well-child exams and provider recommendations represents a limitation that should be addressed in future studies through formal multicollinearity diagnostics.

The findings of this study carry heightened importance for population health and cancer prevention, particularly in the post-pandemic landscape. Starting in 2020, HPV vaccination initiation rates experienced their first stagnation since 2013 [55]. This trend is likely attributable to increased vaccine hesitancy and a decrease in well-child appointment attendance during and after the pandemic. Providers across various specialties consistently recommend the HPV vaccine. Moreover, state and national policies and programs need to enhance their overall quality and adaptability to effectively dismantle the systemic barriers that low-resourced CA adolescents often face. This includes considering the nuanced socioeconomic factors and potentially developing culturally tailored interventions. Finally, CA families should actively partner with their healthcare providers and local governments to participate in medical education activities.

This study identifies significant disparities between CA and aggregated AA populations and within CA populations in sociodemographic factors, healthcare access/utilization, and HPV vaccination rates. Aggregating diverse ethnic groups risks overlooking meaningful disparities, potentially hindering healthcare improvements [56, 57]. Recognizing subgroup differences is vital for data-driven, evidence-based interventions that address the unique needs of communities like CA adolescents [58].

## Data Availability

The NIS-Teens data are available at the CDC Website: https://www.cdc.gov/nchs/nis/data_files_teen.htm. Data requests for restricted access data can be made online through https://www.cdc.gov/rdc/application-process/accessing-restricted-data.html.
